# Effects of Highly Branched Cyclic Dextrin-Based Carbohydrate and Protein Co-Ingestion on Endurance Capacity and Metabolic Response in Soccer Players: A Randomized Crossover Trial

**DOI:** 10.3390/nu18172855

**Published:** 2026-09-01

**Authors:** Ki-Tae Song, Hyuck Lee, Jun-Young Sung, Jin-Kuk Baek, Seong-Won Park, Kyu-Jin Hyun, Eun-hui Jin, Ki-Woong Noh, Sok Park

**Affiliations:** 1 Institute of Exercise Medicine and Science, Kwangwoon University, Seoul 01897, Republic of Korea; 2 Department of Physical Education, Korea National Sport University, Seoul 05541, Republic of Korea; 3 Department of Education, Graduate School, Kwangwoon University, Seoul 05541, Republic of Korea; 4 Department of Physical Education, Graduate School, Kyunghee University, Seoul 17104, Republic of Korea; 5 College of Sport Science, Sungkyunkwan University, Suwon 16419, Republic of Korea

**Keywords:** carbohydrate-protein co-ingestion, carbohydrate supplementation, endurance capacity, highly branched cyclic dextrin

## Abstract

Background/Objectives: Highly branched cyclic dextrin (HBCD) may improve carbohydrate (CHO) availability during soccer-specific intermittent exercise via favorable gastrointestinal properties. Whether co-ingestion with whey protein confers additional ergogenic benefits beyond HBCD alone remains unclear. Methods: Nine male soccer players completed a single-blind, randomized crossover trial (≥7-day washout) with three conditions, Placebo (water), CHO, and isocaloric HBCD plus whey protein isolate (CHOP), during a standardized, ~120-min laboratory-based soccer-specific intermittent exercise protocol (not an actual match). The 500-mL beverage, split into two 250-mL servings, was consumed before exercise and at half-time during the exercise protocol. Respiratory quotient (RQ), substrate oxidation, heart rate, RPE, digestive symptoms, running time, and distance were assessed, with condition effects tested via repeated-measures ANOVA at each cycle. Results: Compared with Placebo, CHO increased RQ (*p* = 0.042) and carbohydrate oxidation (*p* = 0.048) while reducing fat oxidation (*p* = 0.034) at the final cycle, reflecting greater late-exercise CHO utilization without improving endurance performance. Compared with Placebo, CHOP significantly improved both running time (*p* = 0.019) and running distance (*p* = 0.025), with no significant difference from CHO on either outcome. RPE was significantly lower in CHOP than in Placebo during the latter stage of exercise (*p* = 0.016). Conclusions: HBCD ingestion before exercise and at half-time promoted greater late-exercise CHO utilization without increasing cardiovascular or gastrointestinal strain. Co-ingestion of whey protein with HBCD provided ergogenic benefits over Placebo by improving fatigue-limited endurance performance, supporting isocaloric CHOP co-ingestion as a practical nutritional strategy for prolonged soccer-specific intermittent exercise.

## 1. Introduction

Competitive soccer presents a uniquely demanding physiological environment in which players continually oscillate between low- and high-intensity actions, while navigating complex technical and tactical scenarios [1,2]. Over a full match, athletes may perform >1000 changes in locomotor activity, coupled with frequent accelerations, decelerations, and rapid directional shifts, all imposed by the fluid and unpredictable nature of play [3,4]. Although short sprints and high-speed actions contribute minimally to overall distance, these efforts often precede decisive moments such as goal-scoring opportunities or defensive interventions [5,6]. Such repeated non–steady-state demands increase metabolic strain and accelerate glycogen depletion, thus contributing to a decline in high-intensity running, technical accuracy, and cognitive–perceptual function late in matches [7,8]. This underscores the need for nutritional approaches that support carbohydrate (CHO) availability, particularly before exercise and during half-time periods, when substrate replenishment is feasible [9,10].

CHOs are central to sustaining exercise involving repeated fluctuations in intensity because of their capacity for rapid adenosine triphosphate turnover and their essential role in maintaining metabolic homeostasis. Muscle glycogen provides an immediate substrate for contraction, whereas hepatic glycogen stabilizes blood glucose levels and helps prevent central fatigue [11,12]. As exercise intensity increases during high-intensity, glycolytically demanding activity such as repeated sprinting or accelerations, CHO oxidation progressively supersedes fat oxidation because of the elevated glycolytic flux and recruitment of fast-twitch motor units with high glycogenolytic capacity, as directly demonstrated during actual match play, where up to three-quarters of individual muscle fibers become substantially glycogen-depleted by full time [13,14]. When muscle glycogen levels are critically low, athletes lose the ability to sustain repeated high-intensity efforts, while insufficient liver glycogen undermines euglycemia, thereby impairing decision-making and skill execution. Therefore, maintaining CHO availability is fundamental to preventing late-exercise performance decrements, especially in intermittent sports, in which energy demands shift rapidly [15]. Despite strong evidence favoring CHO ingestion, its practical implementation is influenced by gastrointestinal (GI) tolerance, solution osmolality, and gastric emptying. Specifically, conventional CHO beverages may induce GI issues, such as bloating, nausea, or abdominal pain, particularly when consumed in high volumes or concentrations [16,17]. Additionally, in soccer, opportunities for fluid intake during play are limited, making the half-time interval of a match an ingestion window that requires rapid but tolerable consumption of substantial CHO doses [18]. These constraints have stimulated interest in CHO formulations with lower osmotic loads and superior GI compatibility [19].

Highly branched cyclic dextrin (HBCD) has emerged as a promising CHO source that can address these challenges. HBCD, which is produced via the enzymatic cyclization of amylopectin, possesses a high molecular weight and low-dextrose equivalent, thus yielding solutions with reduced osmolality and favorable gastric emptying characteristics [20]. Research has shown that HBCD can attenuate GI symptoms, mitigate fluctuations in blood glucose and lactate, reduce perceived exertion, and enhance endurance performance [21,22]. Moreover, recent studies indicate that HBCD may support metabolic and perceptual responses during resistance exercise [23], which underscores its potential for broader applicability in intermittent high-intensity exercise contexts. Additionally, the co-ingestion of CHOs and protein (CHOP) has been proposed to enhance metabolic responses and mitigate perceived exertion. Protein ingestion may enhance insulin secretion, increase glucose uptake, supply amino acids for energy metabolism and tissue repair, and reduce muscle damage [24]. Accordingly, some studies have reported reduced perceived exertion, improved endurance, and attenuated biomarkers of muscle disruption following the ingestion of CHOP mixtures [25]. However, when total energy and CHO availability have been controlled experimentally, research findings have been inconsistent, which highlights the contextual dependency of protein’s ergogenic effects [26].

Against this backdrop, this study investigates whether isocaloric ingestion of HBCD, either alone or combined with rapidly digestible whey protein, modulates metabolic regulation and fatigue-limited endurance performance during prolonged soccer-specific intermittent exercise (SSIE). Specifically, it aims to determine whether protein co-ingestion (i.e., CHOP) offers additional ergogenic benefits, compared with CHO ingestion alone.

## 2. Materials and Methods

### 2.1. Participants

This study recruited university-level (collegiate) competitive soccer players registered with the Korea Football Association who were currently enrolled at or on official leave from K University (Seoul, Republic of Korea). Recruitment was limited to male athletes affiliated with the university team; female soccer players were not enrolled in this initial trial, consistent with the population available through the recruiting institution. Ten young adult male soccer players participated in this study (*n* = 10). Of these ten randomized participants, one withdrew before completing all three conditions; the remaining nine finished the full crossover protocol and were retained for efficacy analysis, as detailed in Section 3.1. Prior to recruitment, all candidates received a comprehensive explanation of the study’s purpose and procedures, potential risks and benefits, data-protection measures, and their right to withdraw at any time. Subsequently, they provided their written informed consent. Eligibility was confirmed using a pre-participation health questionnaire and a baseline physical examination based on predefined inclusion and exclusion criteria. Inclusion criteria comprised [age 18–25 years, ≥5 years of competitive soccer training, minimum training frequency of 3 sessions/week]. Exclusion criteria comprised [musculoskeletal injury within the preceding 3 months, diagnosed cardiovascular or metabolic disease, use of medications affecting substrate metabolism]. Only participants who met all criteria were included. The study protocol was approved by the institutional review board of K University (IRB No. 7001546-20250207-HR(SB)-001-05, approved on 7 February 2025). Participants’ characteristics are presented in Table 1.

### 2.2. Preliminary Test

During Visit 1 (Week 1), potential participants received a detailed briefing on the study’s purpose and procedures, they provided their written informed consent, and they completed the Physical Activity Readiness Questionnaire. Before the main trials, participants completed a maximum-running-speed test (V_max_) to individualize exercise intensity, while they were briefed on the SSIE protocol. Preparatory guidelines mirrored those subsequently applied before each experimental trial: participants were instructed to refrain from strenuous training for the two days prior, to abstain from alcohol, caffeine, and tobacco for the preceding 24 h, and to report to the laboratory after approximately six hours without food intake. V_max_ testing was performed on a motorized treadmill (T150, COSMED, Rome, Italy) with breath-by-breath gas analysis using a metabolic cart (Quark CPET, COSMED, Rome, Italy). On the day of the test, the participants completed a standardized warm-up activity (jogging at 8 km/h for 5 min, followed by 5 min of stretching). The V_max_ test started at 10 km/h and increased by 1 km/h every minute until volitional exhaustion. V_max_ was defined as the highest speed sustained for ≥1 min and was used solely to individualize the pace of the final run-to-fatigue (RTF) stage (Cycle 7), which was performed at a constant speed equivalent to 80% of each participant’s V_max_ [27].

### 2.3. Experimental Design

The study’s experimental phase required each participant to attend four laboratory sessions, all arranged within the same 2-h window of the day and on the same weekday, aiming to reduce variability associated with circadian fluctuations. A washout interval of at least seven days separated consecutive visits to the clinic. To ensure standardized physiological conditions, participants were asked to avoid any demanding physical exertion for the preceding 48 h; refrain from consuming alcohol, caffeine, or tobacco for 24 h; and arrive at least 6 h after their last meal, regardless of session time of day. Participants were also asked to replicate their usual eating pattern in the 24 h before each visit rather than following a prescribed diet, and this was verbally confirmed upon arrival at each session.

A single-blind randomized crossover design was adopted. The order in which the three nutritional interventions Placebo (water), CHO only, and CHOP were administered was determined for each participant using a computer-generated random-allocation sequence. This sequence was generated and held by a researcher not involved in data collection, concealing each upcoming condition from participants and testing staff until the session began. The six possible condition orderings were distributed as evenly as possible across the cohort. Testing staff were not blinded to treatment order once assigned, so some assessor bias cannot be ruled out, though principal outcomes were captured via automated breath-by-breath and treadmill instrumentation rather than subjective judgment. Upon arrival at the laboratory, participants were asked to rest while seated for a 10-min period, after which they consumed the assigned drink within approximately five minutes, completing intake 15 min before the start of exercise, consistent with procedures used in prior research [28]. Preparation for the exercise protocol involved a standardized warm-up consisting of low-intensity running at 8 km/h for five minutes, followed by an additional five minutes of dynamic stretching. Subsequently, the participants performed the SSIE protocol originally developed by Drust et al. [29]. Throughout the exercise, respiratory and cardiovascular variables, including oxygen uptake (VO_2_), carbon dioxide production (VCO_2_), and heart rate (HR), were continuously captured using a Quark CPET metabolic system (COSMED). Before each testing session, the metabolic analyzer was calibrated for gas concentration, flow, and volume according to the manufacturer’s instructions to ensure measurement fidelity. Subjective and GI responses were assessed at 15-min intervals. The rating of perceived exertion (RPE) was measured using the Borg CR10 scale, whereas digestive symptoms (DS) were assessed using a validated 10-point scale [30,31]. The performance metrics included the total distance covered and the duration for which the participants were able to sustain the exercise. The relative contribution of carbohydrate and fat to overall substrate oxidation expressed as carbohydrate oxidation rate (CHOR) and fat oxidation rate (FOR), respectively, as a percentage of total energy expenditure was calculated from VO_2_ and VCO_2_ using the stoichiometric approach described by Jeukendrup [32]. CHOR and FOR are explicitly defined here as relative percentage contributions to total substrate oxidation, not absolute rates.

### 2.4. Exercise Protocol

**Figure 1:**
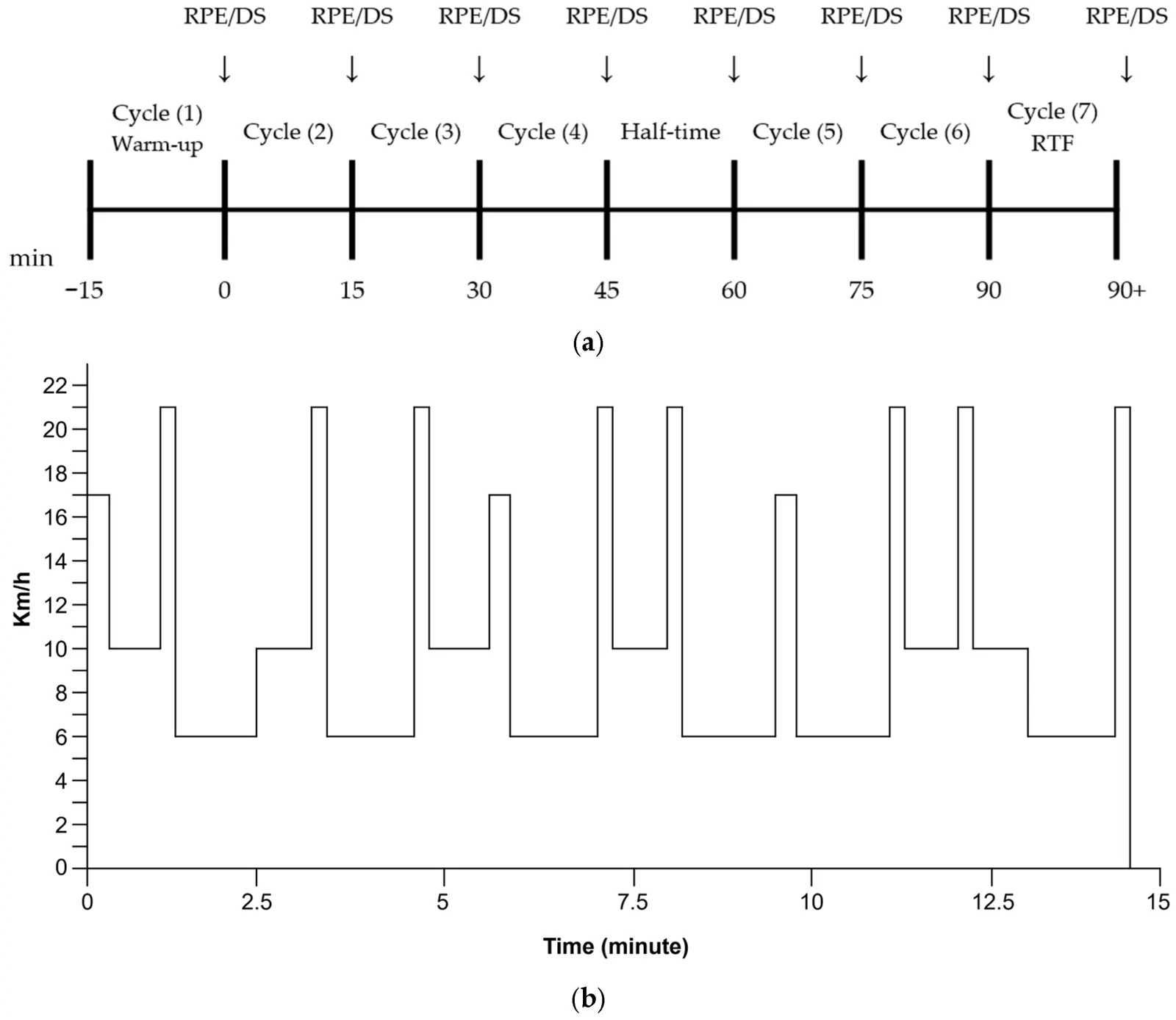
(**a**) SSIE protocol. Participants completed a soccer-specific intermittent exercise (SSIE) protocol consisting of a warm-up, first half, 15-min half-time recovery intervention (Placebo, CHO, or CHOP), second half, and run-to-fatigue test. Downward arrows indicate the assessment time points for RPE and DS between exercise cycles. (**b**) One 15-min cycle (cycle 2 to cycle 6) of soccer-specific intermittent exercise protocol. DS, digestive symptoms; km/h, Kilometer per hour; min, minute; RPE, Rating of perceived exertion; RTF, run-to-fatigue.

The intermittent exercise protocol was structured to closely approximate the temporal and physiological demands of a competitive soccer match, drawing on the framework proposed by Drust et al. [29]. Each session lasted approximately 120 min and included a 15-min preparatory phase, a 45-min first half, a 15-min half-time period, and a second 45-min half. For methodological clarity, the session was divided into seven 15-min segments: Cycle 1 (warm-up), Cycles 2–4 (first half), the half-time recovery interval, Cycles 5–6 (second half), and Cycle 7, which consisted of a RTF (Figure 1a). During Cycles 2–6, all participants completed the same structured SSIE sequence. Each 15-min block consisted of repeated transitions among several locomotor modes performed at fixed treadmill speeds, reflecting typical match-play demands. Specifically, participants alternated between walking at 6 km/h, jogging at 10 km/h, running at 17 km/h, and sprinting at 21 km/h, followed by a brief passive-recovery period lasting 61 s (Figure 1b) [33]. The final segment (Cycle 7 RTF) assessed endurance capacity under fatigue. Participants ran continuously at an intensity corresponding to 80% of their individual V_max_ until volitional exhaustion, and the time to task failure was recorded as the RTF performance outcome [34]. To limit body mass losses associated with dehydration, participants consumed a small bolus of water (2 mL/kg) at the end of each 15-min cycle, consistent with recommendations for intermittent endurance activity [35].

### 2.5. Details of Supplementation

Three supplementation conditions were implemented: Placebo, CHO, and CHOP. Each drink was provided in a total volume of 500 mL and consumed in two portions of 250 mL. Participants ingested the first portion 15 min before beginning exercise and the second at the onset of the half-time interval, with each serving completed within approximately five minutes to ensure consistent timing across visits [36]. The CHO beverage consisted of an 8%-HBCD solution prepared by dissolving 40 g of HBCD (Myprotein, Manchester, UK) in 500 mL of water. For the CHOP condition, the same total volume contained 32 g of HBCD (6.4%) combined with 8 g of whey protein isolate (WPI; SungPoong, Anseong, Republic of Korea). According to the manufacturer’s nutritional information, the CHO and CHOP beverages were energy-matched (152.4 vs. 154.19 kcal, respectively), resulting in a negligible caloric difference of approximately 1.2%. The placebo beverage was plain water (500 mL; 0 kcal). To preserve the single-blind nature of the study, all beverages were standardized for visual appearance, aroma, and flavor using a small, identical quantity of apple-flavored powder concentrate added to each condition, and were dispensed in identical opaque containers (NIF, Food synthetic flavoring, propylene glycol 88.51%, synthetic flavoring). To avoid potential bias, beverage preparation and labeling were performed by an investigator who was not involved in the data collection. Although beverages were standardized for visual appearance, aroma, and volume and dispensed in identical opaque containers, complete masking of taste and mouthfeel particularly between the CHO and CHOP beverages could not be verified, as no formal post-session blinding assessment was performed.

### 2.6. Statistical Analysis

A one-way repeated-measures ANOVA was performed separately at each cycle, with experimental condition (Placebo, CHO, and CHOP) treated as a within-subject factor, to test condition-specific differences at each phase of the exercise protocol. A formal condition × time interaction was not modeled, as our primary interest was condition effects at defined phases (e.g., late-exercise fatigue) rather than the overall time course. The assumption of sphericity was evaluated using Mauchly’s test, and Greenhouse–Geisser corrections were applied when sphericity was violated. Given the small sample size, normality of the within-subject difference scores for each pairwise comparison was also assessed using the Shapiro–Wilk test; where a violation was identified, the corresponding result was cross-checked using the Friedman test as a nonparametric sensitivity analysis. When a significant main effect was detected, Bonferroni-adjusted pairwise comparisons were conducted to identify condition-specific differences. Effect sizes were reported as partial eta squared (partial *η*^2^). A retrospective power analysis (G*Power 3.1.9.7; repeated-measures ANOVA, three conditions, *n* = 9, *r* = 0.5, α = 0.05) indicated 80% power to detect effects of f ≥ 0.465 (partial η^2^ ≥ 0.18). Achieved power for the significant outcomes reported below (partial *η*^2^ = 0.34–0.65) was 0.99–>0.999; however, because post hoc power is mathematically dependent on the observed effect size, this value is reported for transparency only and should not be interpreted as independent evidence of result reliability. Effect estimates with 95% confidence intervals are reported for key outcomes instead. All data are presented as mean ± standard deviation, and statistical significance was set at *p* < 0.05. Statistical analyses were performed using SPSS 25.0 version (IBM Corp., Armonk, NY, USA).

## 3. Results

### 3.1. Participants

**Figure 2:**
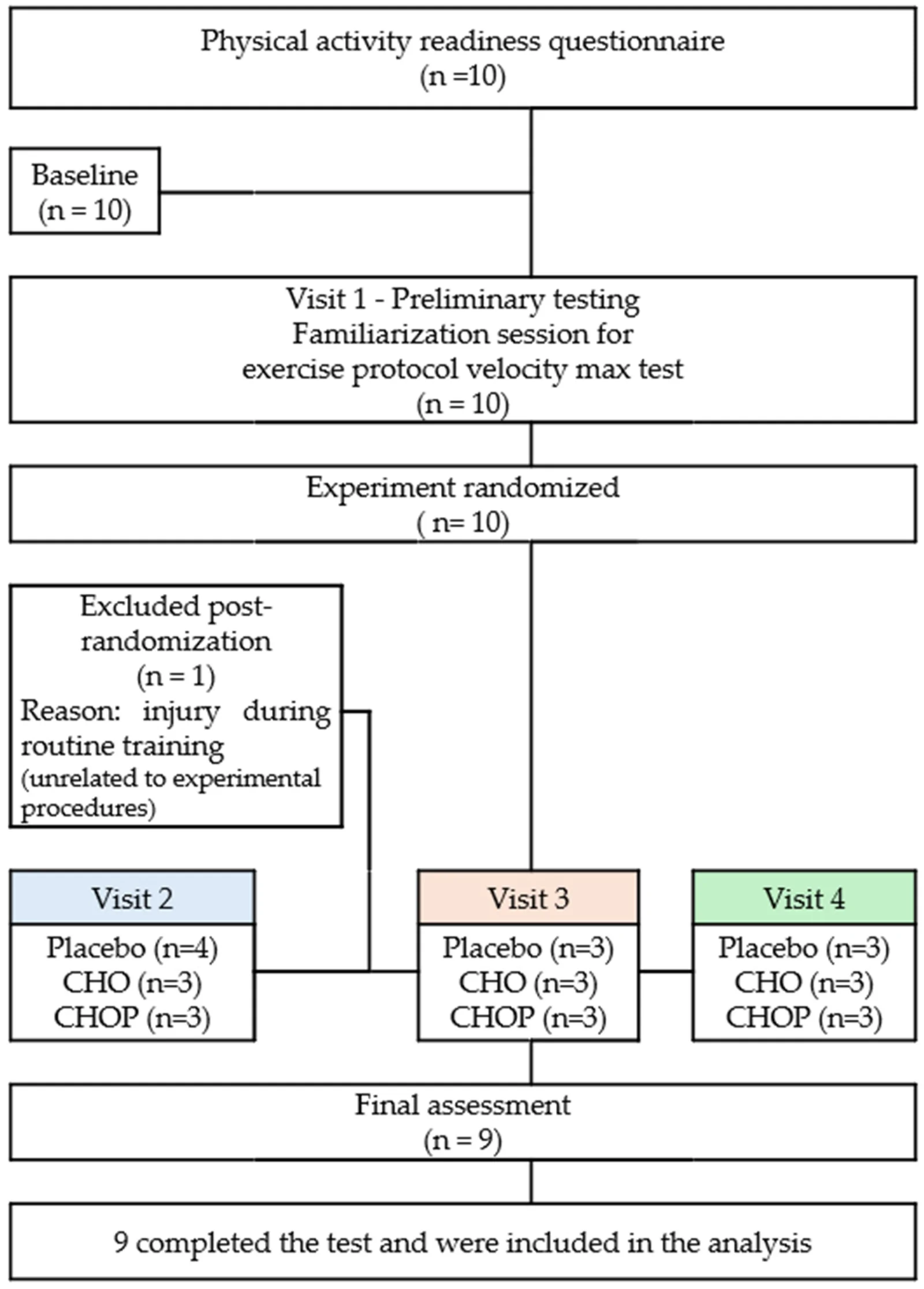
Study flowchart. CHO: carbohydrate; CHOP: carbohydrate and protein.

Ten male collegiate soccer players completed the preliminary testing and familiarization procedures and entered the randomized crossover phase of the study. One participant subsequently withdrew because of injuries sustained during routine training sessions or competitive matches unrelated to the experimental procedures. Withdrawn participants were excluded from the efficacy analysis because they did not complete the full crossover protocol. No adverse events attributable to any beverage condition occurred over the course of the study. Given the constraints of the sample size, formal statistical testing for period or carryover effects associated with treatment order was not undertaken, and this possibility should be borne in mind when interpreting the crossover comparisons. Therefore, data from the 9 participants who completed all 3 experimental conditions (Placebo, CHO, CHOP) were included in the final analyses (Figure 2). Participants’ demographic and anthropometric characteristics are presented in Table 1. Of the 9 athletes included in the final analysis, 2 reported regular smoking habits, defined as approximately 20 cigarettes per week, and 5 reported occasional alcohol consumption, defined as no more than 5 standard drinks per week.

### 3.2. Respiratory Quotient

**Figure 3:**
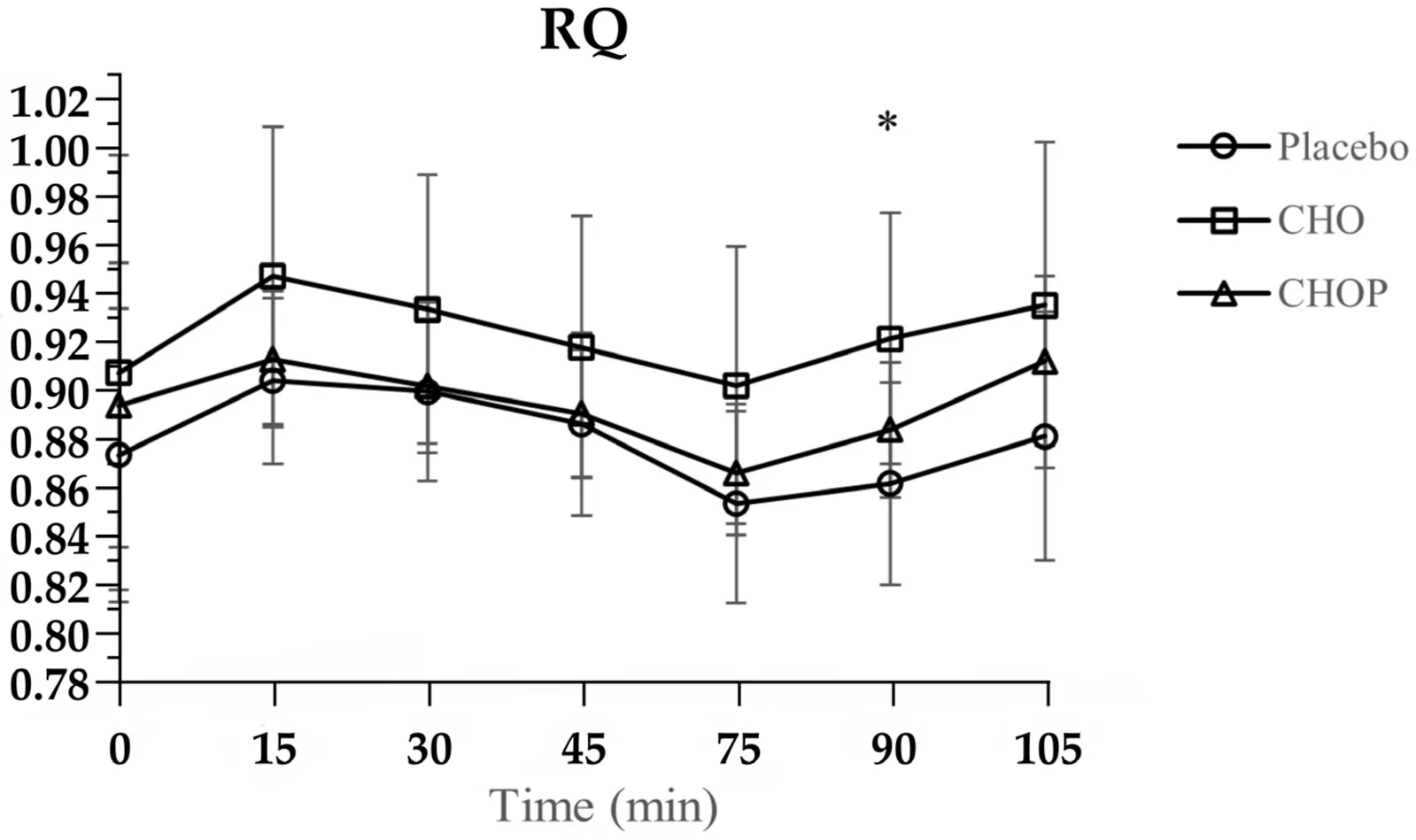
Respiratory quotient during the soccer-specific intermittent exercise protocol across experimental conditions. * *p* < 0.05 for CHO condition versus Placebo at 90 min. RQ, respiratory quotient; CHO, carbohydrate condition; CHOP, carbohydrate and protein.

A significant main effect of condition was observed for RQ at Cycle 6 (*p* = 0.038, partial *η*^2^ = 0.372). Bonferroni-adjusted post hoc comparisons indicated that RQ was significantly higher in the CHO condition than in the Placebo condition (*p* = 0.042) (Table 2 and Figure 3). Difference scores for RQ satisfied the normality assumption across all pairwise comparisons (Shapiro–Wilk *p* = 0.068).

### 3.3. Carbohydrate Oxidation Rate

**Figure 4:**
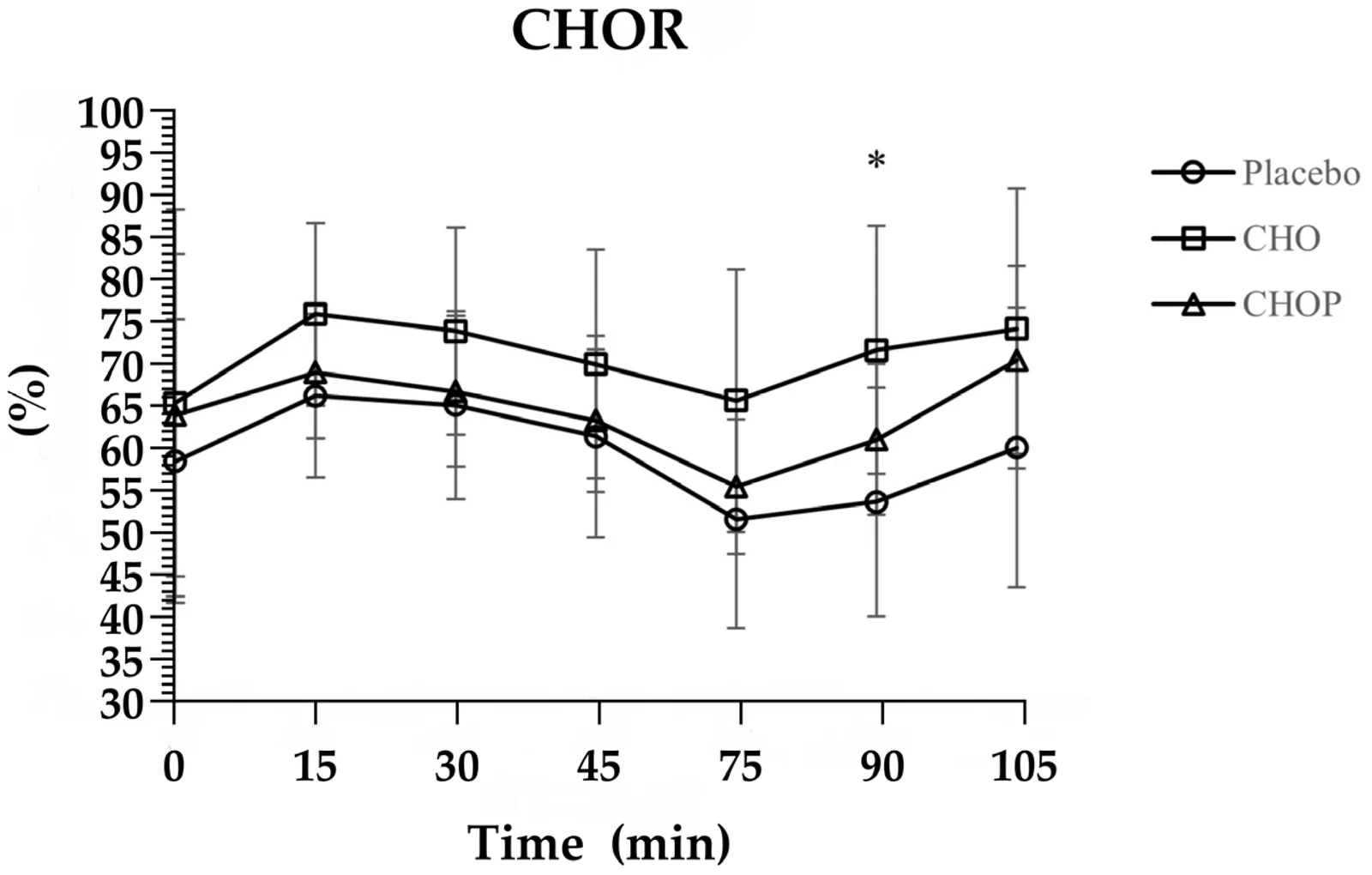
CHOR during the soccer-specific intermittent exercise protocol across experimental conditions. * *p* < 0.05 for CHO versus Placebo at 90 min. CHO, carbohydrate condition; CHOP, carbohydrate and protein; carbohydrate oxidation rate; CHOR, carbohydrate oxidation rate.

A significant main effect of condition was observed for CHOR at Cycle 6 (*p* = 0.037, partial *η*^2^ = 0.337). Bonferroni-adjusted post hoc comparisons indicated that CHOR was significantly higher in the CHO condition than in the Placebo condition (*p* = 0.048) (Table 3 and Figure 4). Difference scores for CHOR satisfied the normality assumption across all pairwise comparisons (Shapiro–Wilk *p* = 0.10).

### 3.4. Fat Oxidation Rate

**Figure 5:**
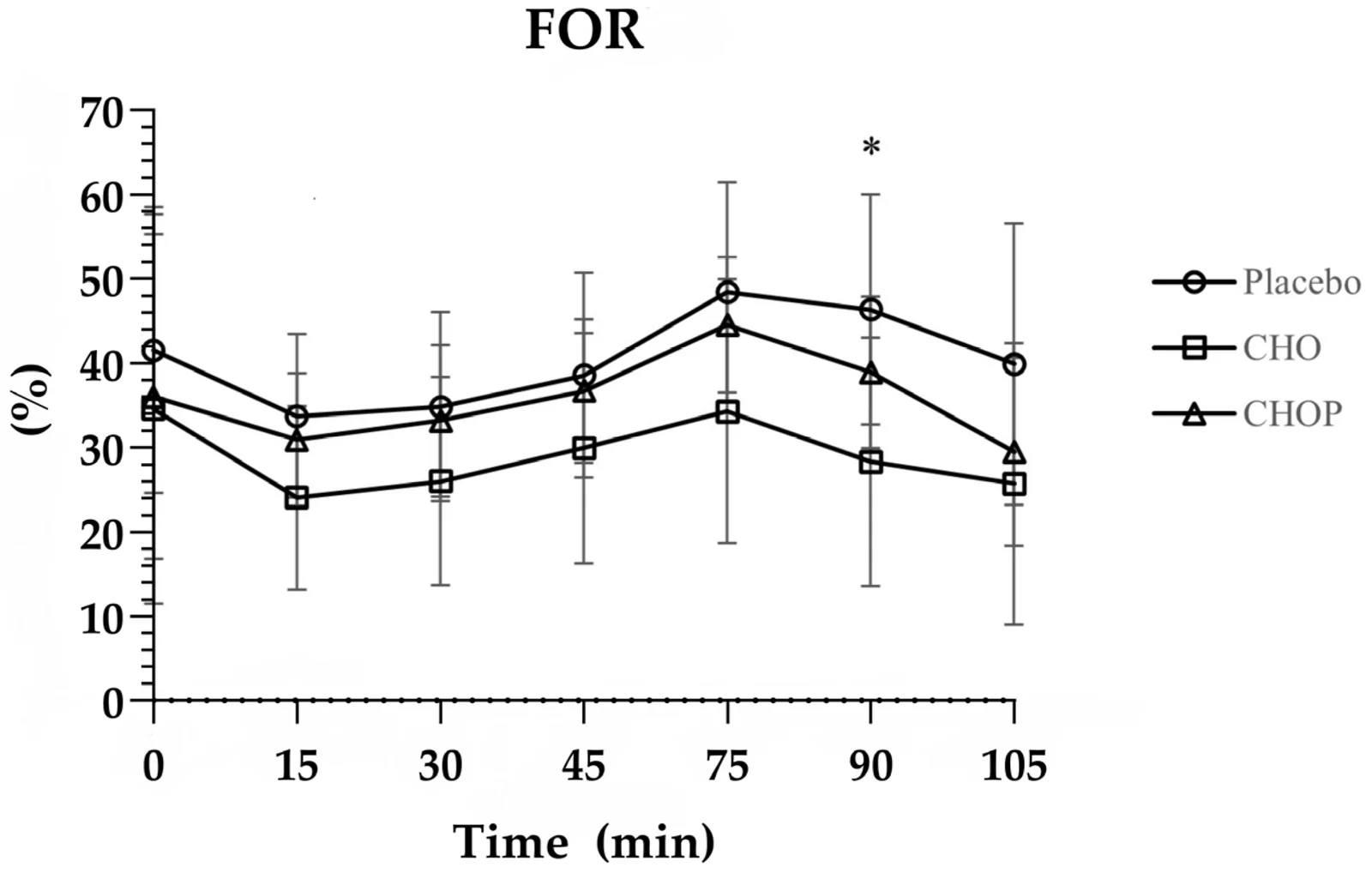
FOR during the soccer-specific intermittent exercise protocol across experimental conditions. * *p* < 0.05 for CHO versus Placebo at 90 min. CHO, carbohydrate condition; CHOP, carbohydrate and protein; FOR, fat oxidation rate.

A significant main effect of condition was observed for FOR at Cycle 6 (*p* = 0.018, partial *η*^2^ = 0.395). Bonferroni-adjusted post hoc comparisons indicated that FOR was significantly higher in the Placebo condition than in the CHO condition (*p* = 0.034) (Table 4 and Figure 5). Difference scores for FOR likewise satisfied the normality assumption across all pairwise comparisons (Shapiro–Wilk *p* = 0.10).

### 3.5. Heart Rate

Heart rate (HR) increased sharply at exercise onset and stabilized across the mid-exercise cycles before rising again during the fatigue run. Despite this physiologically appropriate progression, no between-condition differences emerged at any measurement point (all *p* > 0.05) (Table 5). Difference scores for HR satisfied the normality assumption across all pairwise comparisons (Shapiro–Wilk *p* = 0.11).

### 3.6. Rating of Perceived Exertion

**Figure 6:**
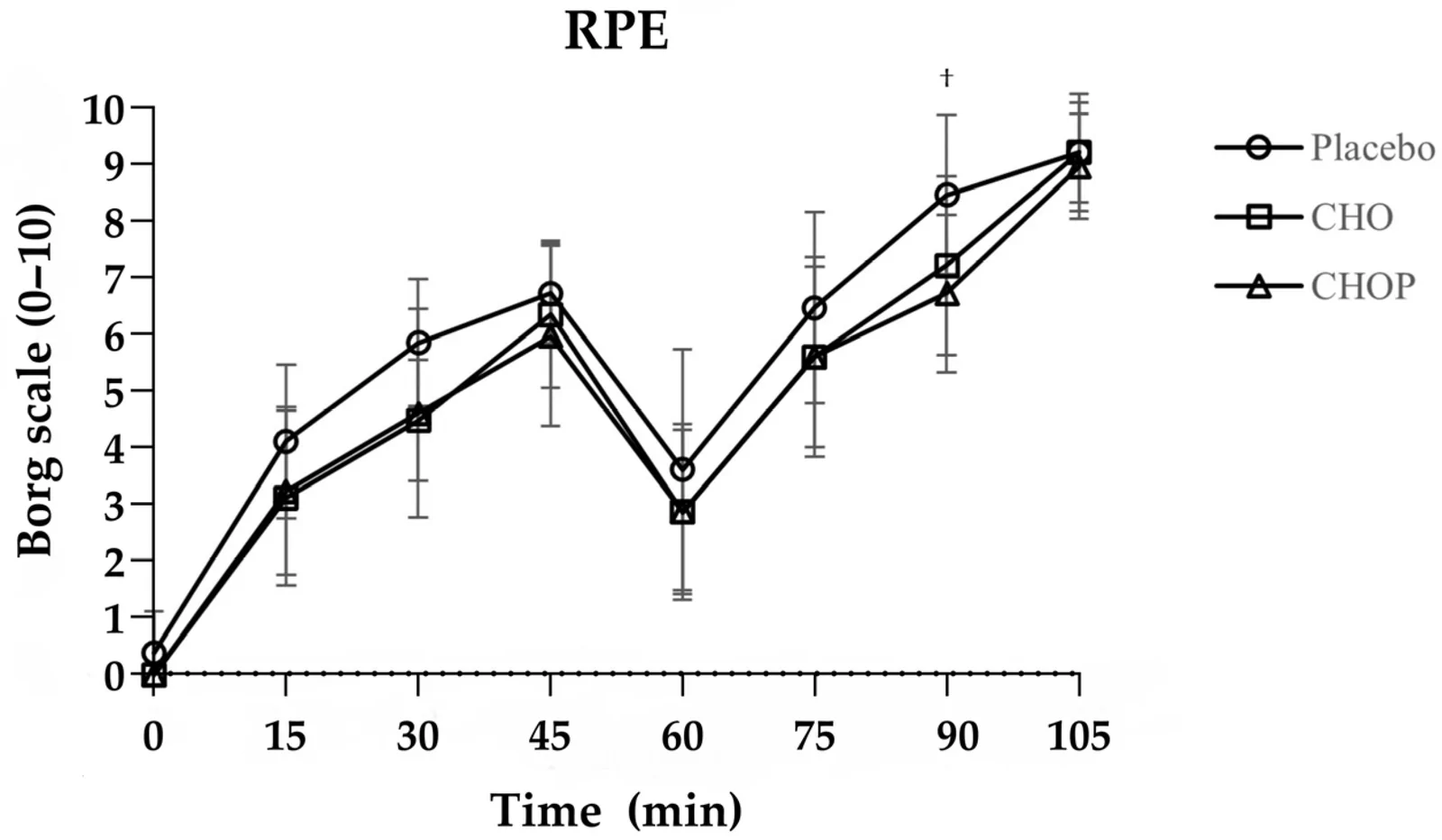
RPE during the soccer-specific intermittent exercise protocol across experimental conditions. † *p* < 0.05 for CHOP versus Placebo at 90 min. CHO, carbohydrate condition; CHOP, carbohydrate and protein; RPE, Rating of perceived exertion.

A significant main effect of condition was observed for RPE at Cycle 6 under the Greenhouse–Geisser correction (*p* = 0.003, partial *η*^2^ = 0.649). Bonferroni-adjusted post hoc comparisons indicated that RPE was significantly higher in the Placebo condition than in the CHOP condition (*p* = 0.016) (Table 6 and Figure 6). Difference scores for RPE deviated from normality in comparisons involving the CHOP condition (Shapiro–Wilk *p* = 0.002–0.016). The Friedman test indicated a significant overall difference among conditions (χ^2^ = 7.00, *p* = 0.030), and the Wilcoxon signed-rank test confirmed a significant difference between CHOP and Placebo (*p* = 0.020).

### 3.7. Digestive Symptoms

GI discomfort remained minimal across all conditions and time points. All participants reported zero DS at Cycle 1, with only trivial increases thereafter. No statistical differences were observed at any cycle (*p* > 0.05), and symptom severity never reached levels indicative of compromised tolerance (Table 7). Difference scores for DS satisfied the normality assumption across all pairwise comparisons (Shapiro–Wilk *p* = 0.19).

### 3.8. Exercise Performance (Running Time and Running Distance)

**Figure 7:**
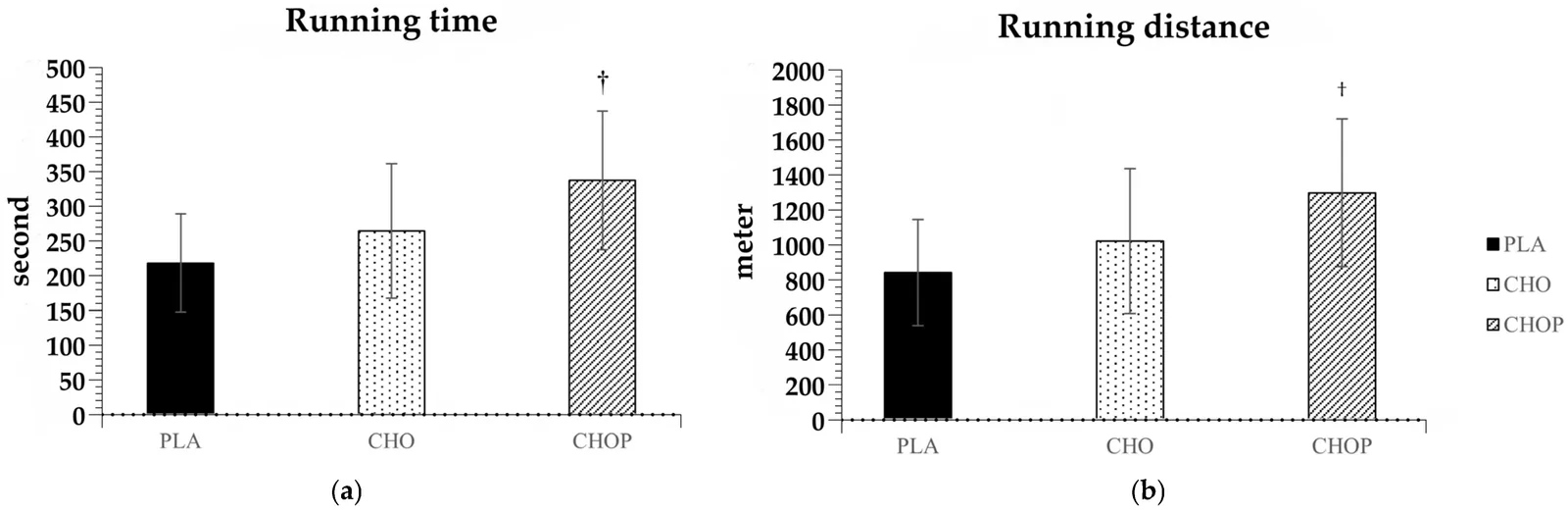
(**a**) Running time during the soccer-specific intermittent exercise protocol across experimental conditions. † *p* < 0.05 for CHOP versus Placebo at cycle 7. (**b**) Running distance during the soccer-specific intermittent exercise protocol across experimental conditions. † *p* < 0.05 for CHOP versus Placebo at cycle 7. CHO, carbohydrate condition; CHOP, carbohydrate and protein; PLA, Placebo.

Running time (RT) showed a significant main effect of condition (*p* = 0.002, partial *η*^2^ = 0.552). Bonferroni-adjusted post hoc comparisons indicated that RT was significantly longer in the CHOP condition than in the Placebo condition (*p* = 0.019). Running distance (RD) showed a significant main effect of condition (*p* = 0.002, partial η^2^ = 0.534). Bonferroni-adjusted post hoc comparisons indicated that RD was significantly greater in the CHOP condition than in the Placebo condition (*p* = 0.025) (Table 8 and Figure 7). Although the raw distributions of RT (Shapiro–Wilk *p* = 0.034, 0.008, and 0.020 for Placebo, CHO, and CHOP, respectively) and RD (*p* = 0.023, 0.008, and 0.015, respectively) deviated from normality within each condition consistent with the characteristic right skew of time-to-exhaustion data. The paired difference scores used in these comparisons were normally distributed (RT: *p* = 0.807, 0.428, and 0.508 for CHO − Placebo, CHOP − Placebo, and CHOP − CHO, respectively; RD: *p* = 0.738, 0.323, and 0.324, respectively), supporting the validity of the parametric approach for these outcomes. Bonferroni-adjusted post hoc comparisons additionally indicated no significant difference between the CHOP and CHO conditions for either RT (mean difference = 89.3 s, 95% CI [−16.4, 195.0], *p* > 0.05) or RD (mean difference = 350.0 m, 95% CI [−58.9, 758.9], *p* > 0.05), and no significant difference between the CHO and Placebo conditions for either outcome (RT: mean difference = 64.6 s, 95% CI [−19.2, 148.3], *p* = 0.340; RD: mean difference = 256.5 m, 95% CI [−77.4, 590.5], *p* = 0.343). The significant improvement in RT and RD was therefore specific to the CHOP-versus-Placebo comparison.

## 4. Discussion

This randomized crossover trial examined whether isocaloric ingestion of HBCD alone or its co-ingestion with WPI before the start of matches and at half-time modulates metabolism, perceptual responses, and endurance capacity during prolonged SSIE. Relative to the Placebo condition, the CHO condition increased the RQ and CHOR while suppressing the FOR late in Cycle 6, indicating a greater reliance on CHOs under high metabolic stress. However, this metabolic shift did not translate into a statistically significant improvement in RTF performance, a finding that, as detailed in the Limitations, should be interpreted in light of the study’s power to detect only large effects. In contrast, CHOP ingestion significantly improved both RT and RD compared with Placebo, despite producing only modest differences in substrate utilization relative to CHO. HR and DS were not significantly altered by beverage type, while DS remained minimal across conditions, underscoring the good tolerance of both HBCD formulations. In contrast, RPE was significantly lower in the CHOP condition than in the Placebo condition during the latter stage of exercise. These findings suggest that (i) HBCD can favor CHO utilization during demanding late-match periods and (ii) adding a small dose of rapidly digestible protein to an isocaloric HBCD beverage may confer an additional advantage for sustaining endurance capacity under soccer-specific fatigue, independent of large changes in cardiorespiratory load or GI comfort.

CHO availability is a key determinant of performance in intermittent high-intensity sports, in which repeated accelerations, sprints, and directional changes impose high glycolytic demands and accelerate glycogen depletion [37,38]. Recent narrative and scoping reviews within the context of soccer have consistently emphasized that sufficient CHO intake before and during matches is crucial for maintaining high-intensity running, technical skill, and cognitive function, particularly in the second half and extra-time periods [39,40].

In the present study, CHO ingestion in the form of an 8%-HBCD solution increased the RQ and CHOR and reduced FOR in Cycle 6, compared with Placebo ingestion. This pattern aligns with the canonical shift toward CHO reliance as the intensity of play and cumulative fatigue increase [37,41]. The greater RQ and CHOR in the CHO condition likely reflect preserved exogenous CHO availability, though blood metabolites were not measured to confirm this. Similar late-exercise benefits of CHO ingestion on substrate use and performance have been reported in simulated soccer-match protocols using higher-concentration CHO–electrolyte solutions (e.g., 12% CHO) [36,39]; the present total CHO dose (~40 g across the session) was lower than in those protocols, which may explain the comparatively modest magnitude of the substrate-utilization shift observed here. The choice of HBCD as a CHO source is relevant, given its unique physicochemical properties. HBCD consists of large, highly branched cyclic molecules derived from amylopectin, producing solutions with low osmolality and relatively rapid gastric emptying, compared with conventional maltodextrin or glucose polymers [22,42]. In prior trials using conventional maltodextrin-based CHO beverages at comparable concentrations, GI symptoms have been commonly reported during intense endurance exercise, particularly bloating and stomach fullness [22]; by contrast, DS scores in the present study remained minimal across all conditions (Section 3.7), consistent with HBCD’s favorable gastric tolerability profile. Experimental research indicates that ingesting HBCD during cycling can attenuate the RPE and modulate blood glucose and lactate responses, compared with maltodextrin [40], while more recent data from CrossFit athletes show that 30 g of cyclodextrin does not markedly change global performance outcomes but may subtly influence neuromuscular fatigue [43]. Our findings partly extend the literature on prolonged soccer-specific intermittent running. The elevation in the RQ and CHOR in Cycle 6 suggests that HBCD can still meaningfully contribute to CHO provision late in exercise, despite the moderate total dose (~40 g across the session). However, the absence of a clear improvement in RTF performance, compared with Placebo ingestion, echoes the equivocal ergogenic effects reported in some HBCD studies, particularly when total CHO availability is modest or when participants begin exercising with adequate glycogen stores [42,43]. Notably, CHOP ingestion did not increase the RQ or CHOR, compared with CHO ingestion, even though it produced significant improvements in both RT and RD. This dissociation between substrate oxidation and performance suggests that mechanisms other than CHO availability may contribute to the CHOP-related performance benefit. The CHOP beverage employed a CHO-to-protein ratio of 4:1 (32 g HBCD to 8 g WPI). Rather than attributing the observed performance benefit to protein-specific mechanisms in isolation, our findings are more consistent with a co-ingestion-specific effect—one that may depend on the combined presence of CHO and protein rather than either macronutrient independently [44,45]. Although the present design cannot directly test these pathways, this pattern is consistent with but does not confirm mechanisms reported elsewhere involving insulin-mediated glucose uptake, amino acid availability, or central fatigue modulation [44,46].

CHOP ingestion is widely advocated in the context of recovery, as it accelerates muscle glycogen resynthesis and enhances muscle protein synthesis, particularly when total CHO intake is suboptimal [44,47]. Meta-analytic and mechanistic studies have demonstrated that adding approximately 0.3–0.4 g/kg of high-quality protein to a CHO beverage can augment insulin secretion, increase glucose uptake, and accelerate glycogen repletion during the early post-exercise window [44,46]. More recent position statements emphasize that endurance athletes can benefit from CHOP ingestion not only post-exercise but also during exercise to support repeated-bout performance and adaptation [48,49]. Although the present study did not include a recovery exercise phase, the RTF test at the end of the protocol functionally resembled a “subsequent bout” performed under cumulative metabolic stress. The superior RTF in the CHOP condition, compared with the Placebo condition, with CHO falling in between, is consistent with studies showing that CHOP beverages can prolong time-to-exhaustion or improve work output in later exercise tasks, even when muscle glycogen content is similar between conditions [45,50]. Dahl et al. reported improved cycling time-to-exhaustion after 5 h of recovery when protein was added to a post-exercise CHO beverage, despite comparable glycogen restoration, which suggests that non-glycogen mechanisms e.g., reduced muscle damage, altered neuromuscular function, or changes in central perception of effort may be involved [45].

Regarding insulin and glycemic regulation, CHOP ingestion is known to potentiate insulin responses and improve glycemic stability in both athletic and clinical populations [51]. A more stable glycemic profile during the later stages of intermittent exercise may help sustain cerebral glucose availability and mitigate central fatigue, thereby supporting the capacity to sustain high-intensity running without necessarily altering the whole-body RQ.

As for amino acid availability and neuromuscular function, WPI provides rapidly absorbed essential amino acids, including leucine and branched-chain amino acids, which support muscle-protein turnover and may attenuate exercise-induced muscle damage and soreness [52,53]. While the acute 8-g dose of WPI used in the present study was modest, repeated exposure in pre-test and half-time windows may still provide sufficient aminoacidemia to subtly influence excitation–contraction coupling or muscle membrane integrity during the final RTF phase of the exercise, though this remains speculative in the absence of direct neuromuscular or membrane-integrity markers in this study.

Regarding the perceptual and central mechanisms of pain, several studies have reported a reduced RPE following CHOP ingestion during or after strenuous exercise, even in the absence of large changes in objective performance [50,54]. In the present study, RPE was significantly lower in the CHOP condition than in the Placebo condition during the latter stage of exercise. This finding proved robust to a nonparametric cross-check despite a normality violation in the underlying difference scores (Section 3.6), lending additional confidence to the result. This reduction in perceived exertion may have contributed to the prolonged RTF performance observed following CHOP ingestion.

Pertaining to CHO availability, contemporary reviews have highlighted that protein co-ingestion appears most ergogenic when total CHO intake is moderate, rather than maximized, and when exercise duration or frequency imposes repeated stress within a single day or a short microcycle [46,47]. In the present study, total CHO provision (~40 g of HBCD) was lower than the typical match-day guidelines (often 30–60 g/h during play) [38,40]. Under such “moderate-CHO” conditions, adding protein may have amplified the functional benefits of the available CHO, yielding a detectable performance advantage over the Placebo condition, even though the CHO condition did not. Collectively, these mechanistic considerations suggest that the CHOP condition may have created a more favorable metabolic and neuromuscular milieu for sustaining late-exercise running capacity, compared with the CHO or Placebo conditions, despite similar external workloads and global cardiovascular responses.

Against this mechanistic background, the specific pattern observed in the terminal RTF test provides additional insights. Although not statistically significant, the ~46-s (21%) longer RTF time observed with CHO relative to Placebo represents a magnitude that could be practically meaningful in a match context, where even brief extensions in fatigue-limited running capacity may influence late-match or injury-time performance. This trend therefore warrants consideration as a potentially relevant, rather than negligible, effect of CHO ingestion alone. In our study, the CHOP condition produced significant improvements in both time-to-exhaustion and running distance, suggesting that CHOP ingestion may exert its strongest effects when athletes transition from intermittent loading to a continuous, fatigue-limited task. Similarly, recent studies have reported that CHOP-ingestion strategies can enhance performance or preserve exercise capacity under repeated or prolonged stress, particularly when total CHO intake is moderate rather than maximal [55,56,57]. These benefits likely arise from the combined influence of more stable glycemia, attenuation of exercise-induced muscle damage, and improved recovery of contractile function, as supported by contemporary studies on protein and amino acid supplementation in endurance athletes [58,59]. The RTF and distance patterns observed in this study’s CHOP condition are best interpreted as preliminary evidence of enhanced fatigue resistance, while acknowledging the limited statistical power of its small sample. These performance-oriented observations provide a useful bridge to the subsequent consideration of perceptual and GI responses, which ultimately constrain the on-field feasibility of HBCD- and CHOP-based strategies.

An important practical observation is that DS remained negligible in all conditions. This aligns with earlier reports showing that HBCD solutions, owing to their low osmolality and rapid gastric emptying, typically induce fewer DS, compared with glucose–maltodextrin beverages, at similar CHO loads [22,42]. Similarly, cyclodextrin ingestion has demonstrated high tolerability during repeated high-intensity CrossFit sessions [43], further supporting the suitability of this CHO for intermittent exercise. The inclusion of 8 g of WPI in the CHOP beverage did not aggravate DS, consistent with evidence that modest doses of rapidly digestible protein can be co-ingested with CHOs without increasing DS when solution osmolality is properly controlled [47,48].

Beyond GI tolerance, perceptual responses showed an additional, noteworthy pattern. The CHOP condition elicited significantly lower ratings of perceived exertion than the Placebo condition during the latter stage of exercise. Contemporary psychobiological models conceptualize perceived effort as an emergent construct shaped by motor command signals, afferent feedback, cognitive appraisal, and affective state, rather than a simple reflection of cardiovascular load [60]. Within this framework, modest improvements in glycemic stability, circulating amino acid levels, or muscle comfort can meaningfully influence perceptual strain at a fixed external workload. Earlier studies have reported that CHOP beverages may reduce perceived exertion and muscle soreness during prolonged or repeated exercise [54,61]. Although insulin and amino acid availability were not measured in the present study, the WPI dose employed is hypothesized to have contributed to these perceptual benefits, consistent with mechanisms proposed in the cited literature; this interpretation remains speculative in the absence of direct biochemical confirmation. Such subtle reductions in perceived exertion, even when not statistically detectable in a small sample, may partly explain the extended RTF observed in the CHOP condition [62]. Nonetheless, because complete sensory masking between beverages was not formally verified, a residual expectancy or taste-related effect on RPE cannot be entirely excluded and should be considered alongside the physiological explanations above.

From a practical standpoint, a moderate dose of HBCD shifted substrate use toward CHOs during late high-intensity running, with no significant between-condition difference in HR or digestive symptoms, suggesting no evidence of increased cardiovascular or GI strain relative to Placebo, supporting the use of low-osmolality HBCD formulations for athletes who struggle with concentrated, high-osmolality CHO beverages (e.g., conventional maltodextrin or glucose-based solutions) within soccer’s limited within-match fluid intake windows [22,40,42,63]. Adding a small amount of WPI to an isocaloric HBCD beverage further extended fatigue-limited running time and lowered perceived exertion under fatigue, suggesting that modest protein inclusion at half-time may support late-match endurance when daily CHO and energy requirements are already met [44,45].

However, these interpretations must be tempered by methodological constraints. The main limitation of this study is its small sample size (*n* = 9). Retrospective power analysis showed the design was adequately powered (0.99–>0.999) to detect the large effects underlying the significant findings but was sensitive only to effects of f ≥ 0.465; detecting a medium effect (f = 0.25) would require ~28 participants. This likely explains the non-significant ~46-s (21%) longer RTF time in the CHO versus Placebo condition (Table 8), which may reflect a true but underpowered effect rather than a null result. Comparable crossover trials examining CHOP co-ingestion and endurance performance have similarly enrolled 9–10 participants after accounting for attrition [45,55], indicating that the present sample size is consistent with what is typically achievable in this highly select athletic population, rather than an isolated limitation of this study’s design. Future studies should determine sample size a priori based on these effect sizes and report 95% confidence intervals alongside point estimates. With only nine participants across six possible condition orderings, sequence balance was necessarily imperfect, so residual period or carryover effects cannot be fully ruled out. Outcomes were also compared across multiple cycles without a family-wise correction, so these cycle-by-cycle results should be read as exploratory rather than confirmatory. Additionally, the RTF test was positioned after the second half to reflect the ecologically relevant late-match/injury-time period, but the resulting high cumulative fatigue may have masked more subtle CHO-related benefits detectable earlier in exercise. Furthermore, this study did not confirm whether volitional exhaustion coincided with attainment of VO_2_max or with elevated blood lactate, precluding differentiation between true metabolic limitation and centrally mediated fatigue. Moreover, the nutritional dosing used was conservative; higher absolute CHO intake, alternative CHOP ratios, or additional ingestion points may elicit more pronounced effects. Direct comparisons of HBCD with other CHO matrices would clarify whether HBCD’s advantages extend beyond GI tolerance. Furthermore, the absence of biochemical and neuromuscular markers restricts mechanistic insights, underscoring the need for studies to incorporate blood sampling, neuromuscular assessments, and extended match simulations. Although treadmill-based SSIE enables strong workload control, it lacks the contextual features of real play. Integrating HBCD and CHOP strategies into small-sided games or competitive fixtures using wearable-based load and skill metrics would enhance these outcomes’ ecological validity. It should also be acknowledged that this trial was registered after recruitment had already begun (KCT0011559, registered 2 February 2026); although the reported outcomes match those specified in the approved study protocol, prospective registration would have provided stronger assurance against selective outcome reporting. Despite these limitations, the present trial provides novel evidence on isocaloric HBCD and HBCD–protein ingestion within a soccer-specific intermittent framework and offers a foundation for refining nutritional strategies that support soccer players’ late-exercise performance under match-simulated conditions.

## 5. Conclusions

This study investigated whether isocaloric ingestion of HBCD, either alone or in combination with WPI, alters metabolism and endurance performance during prolonged soccer-specific intermittent running. Relative to water ingestion, HBCD ingestion before exercise and at the simulated half-time interval promoted greater late-exercise CHO reliance, as indicated by a higher RQ and increased CHOR in the latter stage of the second half; however, it did not significantly improve fatigue-limited endurance performance. In contrast, CHOP ingestion significantly improved both RT and RD compared with Placebo, despite only modest differences in whole-body substrate utilization relative to CHO alone. HR and DS were similar across all conditions, whereas RPE was significantly lower in the CHOP condition than in the Placebo condition, confirming the good tolerability of both HBCD-based drinks. In practical terms, these findings support a specific, actionable strategy: soccer players may benefit from consuming approximately 250 mL of a 6.4–8% HBCD beverage before the exercise and an additional 250 mL containing 8 g of whey protein isolate at the simulated half-time interval, a regimen that supported CHO utilization without increasing cardiovascular or GI strain and additionally improved fatigue-limited endurance capacity relative to HBCD alone. Nonetheless, this study’s small, homogeneous sample, the absence of blood-based mechanistic markers, and its treadmill-based simulations limit the generalizability of these findings. Future research should prioritize two directions arising directly from the present results: first, mechanistic trials incorporating blood glucose, insulin, lactate, and muscle-damage markers are needed to test whether the performance benefit of CHOP over HBCD alone is genuinely attributable to a co-ingestion-specific pathway, as hypothesized here, rather than to CHO availability alone; second, trials should examine whether earlier placement of a fatigue-resistance test within a match-simulation protocol reveals ergogenic benefits of CHO alone that may have been masked by cumulative fatigue in the present design. Extending this line of research to female, elite-level, and non-soccer intermittent-sport athletes will further clarify the generalizability of CHOP co-ingestion as a nutritional strategy.

## Figures and Tables

**Table 1:** Participants’ (*n* = 10) characteristics.

	*n*	Minimum	Maximum	Mean	Standard Deviation
Age	10	18	23	21	±1.77
Body Weight (kg)	10	66	76	70.13	±4.02
Height (cm)	10	167	184	174	±4.96
BMI (kg/m^2^)	10	21.80	24.82	23.17	±1.12
V_max_ (km/h)	10	16	19	17.13	±0.99

BMI, body mass index; V_max_, maximal treadmill running speed (velocity max).

**Table 2:** Descriptive statistics for RQ by cycle and condition.

RQ	Cycle (min) (Mean ± SD)						
	Cycle 1 (0)	Cycle 2 (15)	Cycle 3 (30)	Cycle 4 (45)	Cycle 5 (75)	Cycle 6 (90)	Cycle 7 (RTF)
Placebo	0.8765 ±0.0604	0.9069 ±0.0342	0.9025 ±0.0369	0.8892 ±0.0377	0.8566 ±0.0408	0.8647 ±0.0415	0.8842 ±0.0512
CHO	0.9104 ±0.0893	0.9503 ±0.0611	0.9365 ±0.0552	0.9207 ±0.0539	0.9051 ±0.0571	0.9244 * ±0.0517	0.9382 ±0.0672
CHOP	0.897 ±0.0584	0.9158 ±0.0281	0.905 ±0.0276	0.8936 ±0.0261	0.869 ±0.0254	0.8869 ±0.0278	0.9152 ±0.0348

Values are presented as mean ± SD; *n* = 9. Bonferroni’s test was used to calculate *p* values. CHO *, *p* < 0.05 compared with Placebo condition. C, cycle; CHO, carbohydrate; CHOP, carbohydrate plus protein; RQ, respiratory quotient; RTF, run-to-fatigue; SD, standard deviation.

**Table 3:** Descriptive statistics for CHOR by cycle and condition.

CHOR	Cycle (min) (Mean ± SD)						
	C1 (0)	C2 (15)	C3 (30)	C4 (45)	C5 (75)	C6 (90)	C7 (RTF)
Placebo	58.26 ±16.91	66.07 ±9.72	64.97 ±11.17	61.24 ±2.05	51.33 ±12.99	53.44 ±13.66	59.91 ±16.67
CHO	65.23 ±23.07	75.79 ±10.87	73.81 ±12.35	69.88 ±13.66	65.53 ±15.65	71.53 * ±14.77	74.09 ±16.66
CHOP	63.78 ±19.22	68.88 ±7.89	66.63 ±8.99	63.12 ±8.54	55.25 ±8.02	60.89 ±9.01	70.35 ±11.18

Values are presented as mean ± SD; *n* = 9. Bonferroni’s test was used to calculate *p* values. CHO *, *p* < 0.05 compared with Placebo condition. C, cycle; CHO, carbohydrate; CHOP, carbohydrate plus protein; CHOR, carbohydrate oxidation rate; RTF, run-to-fatigue; SD, standard deviation.

**Table 4:** Descriptive statistics for FOR by cycle and condition.

FOR	Cycle (min) (Mean ± SD)						
	C1 (0)	C2 (15)	C3 (30)	C4 (45)	C5 (75)	C6 (90)	C7 (RTF)
Placebo	41.74 ±16.91	33.93 ±9.72	35.03 ±11.17	38.76 ±12.15	48.67 ±12.99	46.56 ±13.66	40.09 ±16.67
CHO	34.77 ±23.07	24.21 ±10.87	26.19 ±12.35	30.12 ±13.66	34.47 ±15.65	28.48 * ±14.77	25.91 ±16.66
CHOP	36.22 ±19.22	31.12 ±7.89	33.37 ±7.89	36.88 ±8.54	44.75 ±8.02	39.11 ±9.01	29.65 ±11.18

Values are presented as mean ± SD; *n* = 9. Bonferroni’s test was used to calculate *p* values. CHO *, *p* < 0.05 compared with Placebo condition. C, cycle; CHO, carbohydrate; CHOP, carbohydrate plus protein; FOR, fat oxidation rate; RTF, run-to-fatigue; SD, standard deviation.

**Table 5:** Descriptive statistics for HR by cycle and condition.

HR	Cycle (min) (Mean ± SD)						
	C1 (0)	C2 (15)	C3 (30)	C4 (45)	C5 (75)	C6 (90)	C7 (RTF)
Placebo	88.13 ±10.41	146.39 ±13.55	152.12 ±14.94	155.4 ±15.38	151.37 ±14.94	157.04 ±16.26	175.09 ±12.04
CHO	84.32 ±11.11	142.89 ±16.97	152.05 ±18.48	156.84 ±19.08	150.26 ±18.37	155.80 ±19.81	176.84 ±11.09
CHOP	88.58 ±10.58	142.89 ±12.27	151.61 ±14.23	156 ±15.52	150.27 ±15.21	155.99 ±15.85	179.97 ±9.75

Values are presented as mean ± SD; *n* = 9. C, cycle; CHO, carbohydrate; CHOP, carbohydrate plus protein; HR, heart rate; RTF, run-to-fatigue; SD, standard deviation.

**Table 6:** Descriptive statistics for RPE by cycle and condition.

RPE	Cycle (min) (Mean ± SD)							
	C1 (0)	C2 (15)	C3 (30)	C4 (45)	Half-Time (60)	C5 (75)	C6 (90)	C7 (RTF)
Placebo	0.38 ±0.74	4.13 ±1.36	5.88 ±1.12	6.75 ±0.89	3.63 ±2.13	6.50 ±1.69	8.50 ±1.41	9.25 ±0.89
CHO	0	3.13 ±1.55	4.5 ±1.07	6.38 ±1.30	2.88 ±1.55	5.63 ±1.77	7.25 ±1.58	9.25 ±1.04
CHOP	0	3.25 ±1.49	4.63 ±1.85	6 ±1.60	2.88 ±1.46	5.63 ±1.6	6.75 * ±1.39	9 ±0.93

Values are presented as mean ± SD; *n* = 9. * *p* < 0.05 for CHOP versus Placebo. C, cycle; CHO, carbohydrate; CHOP, carbohydrate plus protein; RPE, rating of perceived exertion; RTF, run-to-fatigue; SD, standard deviation.

**Table 7:** Descriptive statistics for DS by cycle and condition.

DS	Cycle (min) (Mean ± SD)							
	C1 (0)	C2 (15)	C3 (30)	C4 (45)	Half-Time (60)	C5 (75)	C6 (90)	C7 (RTF)
Placebo	0	0.38 ±0.74	1.50 ±2.51	1 ±2.14	0.38 ±0.74	1.50 ±3.12	2.13 ±3.4	2.25 ±2.77
CHO	0	0.75 ±1.49	0.88 ±1.64	1.13 ±1.25	0.25 ±0.46	1.88 ±2.7	1.13 ±1.89	1.50 ±1.69
CHOP	0	0.75 ±1.04	1.38 ±2.39	2 ±1.93	0.75 ±1.17	1.88 ±2.59	2.25 ±2.66	2.75 ±3.69

Values are presented as mean ± SD; *n* = 9. C, cycle; CHO, carbohydrate; CHOP, carbohydrate plus protein; DS, Digestive symptoms; RTF, run-to-fatigue; SD, standard deviation.

**Table 8:** Descriptive statistics for exercise performance by cycle and condition.

	Placebo	CHO	CHOP
Running time (sec)	218.88 ± 71.04	265.38 ± 97.19	338.13 ± 99.94 †
Running distance (meter)	840.97 ± 304.37	1020.92 ± 416.57	1297.47 ± 423.37 †

Values are presented as mean ± SD; *n* = 9. Bonferroni’s test was used to calculate *p* values. CHOP †, *p* < 0.05 compared with Placebo condition. CHO, carbohydrate; CHOP, carbohydrate plus protein; SD, standard deviation.

## Data Availability

The datasets generated and/or analyzed during the current study are available in the Figshare repository: https://doi.org/10.6084/m9.figshare.30738956.

## Abbreviations

ANOVA	Analysis of variance
BMI	Body mass index
CHO	Carbohydrate
CHOP	CHO and protein
CHOR	Carbohydrate oxidation rate
CRIS	Clinical Research Information Service
DS	Digestive symptoms
FOR	Fat oxidation rate
GI	Gastrointestinal
HR	Heart rate
km/h	Kilometers per hour
min	Minutes
PAR-Q+	Physical Activity Readiness Questionnaire for Everyone
RD	Running distance
RPE	Rating of perceived exertion
RQ	Respiratory quotient
RT	Running time
RTF	Run-to-fatigue
s	Seconds
SD	Standard deviation
SSIE	Soccer-specific intermittent exercise
VCO_2_	Carbon dioxide production
VO_2_	Oxygen consumption
V_max_	Maximum velocity
WHO	World Health Organization
